# Using A Google Web Search Analysis to Assess the Utility of ChatGPT in Stem Cell Therapy

**DOI:** 10.1093/stcltm/szad074

**Published:** 2023-11-04

**Authors:** Long Chen, Hui Li, Yiqi Su, Zhen Yang, Zihao He, Du Wang, Jiao Jiao Li, Dan Xing

**Affiliations:** Arthritis Clinic and Research Center, Peking University People’s Hospital, Peking University, Beijing, People’s Republic of China; Arthritis Clinic and Research Center, Peking University People’s Hospital, Peking University, Beijing, People’s Republic of China; Arthritis Clinic and Research Center, Peking University People’s Hospital, Peking University, Beijing, People’s Republic of China; Arthritis Clinic and Research Center, Peking University People’s Hospital, Peking University, Beijing, People’s Republic of China; Arthritis Clinic and Research Center, Peking University People’s Hospital, Peking University, Beijing, People’s Republic of China; Arthritis Clinic and Research Center, Peking University People’s Hospital, Peking University, Beijing, People’s Republic of China; School of Biomedical Engineering, Faculty of Engineering & IT, University of Technology Sydney, Sydney, NSW, Australia; Arthritis Clinic and Research Center, Peking University People’s Hospital, Peking University, Beijing, People’s Republic of China

**Keywords:** stem cell therapy, ChatGPT, Google, utility, web search

## Abstract

**Objective:**

Since its introduction, the use of ChatGPT has increased significantly for medically related purposes. However, current research has not captured its applications in providing information on stem cell therapy. To address this gap, the present study compared the effectiveness of ChatGPT to Google in answering medical questions related to stem cell therapy.

**Methods:**

The search term “stem cell therapy” was used to perform a Google web search, and the top 20 frequently asked questions along with answers were recorded together with relevant website sources. Of these questions, the top 10 questions were separately entered into ChatGPT, and the answers and the sources were recorded. Then, the following statement was entered into ChatGPT: “Do a Google search with the search term ‘stem cell therapy’ and record 20 common questions related to the search term.” After obtaining these questions, each question was separately entered into ChatGPT for an answer and source.

**Results:**

A majority of the top 20 questions provided by Google were related to fact, whereas a majority of the questions provided by ChatGPT were related to policy. The answer sources used by Google were mostly drawn from medical practice, while those used by ChatGPT were mostly drawn from academic information.

**Conclusion:**

Compared to Google, ChatGPT exhibits stronger capabilities in promoting awareness of stem cell therapy. ChatGPT has the ability to eliminate misleading information by providing accurate and reliable answers. However, the responses provided by ChatGPT are still general in nature and cannot substitute academic sources for providing specialized knowledge.

## Graphical Abstract

**Graphical Abstract:**
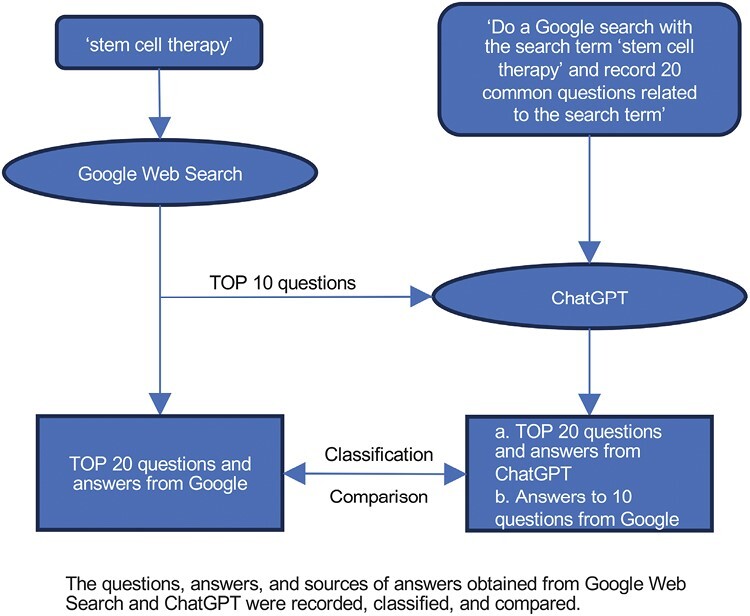

**Significance Statement**

The study concluded that ChatGPT has stronger capabilities in patient education and promoting awareness of stem cell therapy due to its ability to eliminate false or misleading information and provide reliable and accurate answers. It is also worth noting that the results demonstrated the significant impact of ChatGPT in patient education, mitigating misuse, and enhancing doctor-patient communication within the domain of stem cell therapy.

## Introduction

Since its introduction in late 2022, ChatGPT has instilled confidence in the future of artificial intelligence, particularly in applications involving sourcing of information.^1^ As the latest advancement in deep learning, it is a generative pretraining converter based on GPT-3.5 architecture. Like many Large Language Models (LLM), ChatGPT can generate text in various styles and for different purposes, but with significantly greater precision, detail, and coherence. Its widespread use is due to its exceptional natural language processing power, which effectively simulates human interactions. With extensive training on a large corpus of texts, it can produce responses similar to those generated by humans.^1^ In just a few months, the use of ChatGPT in the medical field has been extensively studied. Its potential uses range from assisting students with academic writing to aiding software engineers in coding. Furthermore, scientists in the medical field are exploring the potential applications of ChatGPT in clinical work and research. In contrast to other LLMs, ChatGPT has been described by numerous studies as a promising and even revolutionary tool for medical academic writing and scientific research.^2,3^

GPT-based chatbots such as ChatGPT are useful tools for extracting information from electronic medical records and assisting with literature retrieval. Authors of medical academic papers can utilize ChatGPT by inputting their requirements and tips into the chat box to receive a draft text of their intended article, which they can then review and edit as necessary. Once the editing process is complete, ChatGPT can also perform automated review of the article and provide guidance on academic writing formats.^3,4^ Moreover, ChatGPT is an efficient tool for clinicians seeking to provide and summarize treatment guidelines for specific diseases, including information on the potential benefits and harms of various treatment options, side effects, and drug interactions, ultimately facilitating decision-making.^5,6^ Another example of medically related applications involves using ChatGPT to summarize patients’ discharge medical records based on a given template, which has potential to significantly improve the daily work efficiency of clinicians and reduce their workload.^7^

Recently, there has been a significant increase in research on stem cell therapy for treating a variety of diseases, leading to advances in cell therapy methods that have generated high expectations.^8^ Stem cell therapy has been extensively researched across various fields and has shown potential clinical applications. Neural stem cells, for example, are being considered as a promising therapy to enhance treatment for Alzheimer’s disease and other neurodegenerative diseases.^9^ However, while some stem cell-based treatments have been approved by the FDA, many other interventions involving stem cells are still in the clinical trial stage.^10,11^ Stem cell therapy is a relatively new treatment method for the majority of diseases it has been applied to, and its safety and efficacy has been captured in a myriad of information sources on the internet with varying quality. This has generated myths about stem cell therapy, as well as overly positive or negative anticipations of its effectiveness, among the general public who often have limited knowledge and understanding in the topic area. The emergence of ChatGPT may provide an excellent opportunity to improve public education on the benefits and potential harms of stem cell therapy based on available information sources.

ChatGPT has been recognized for its potential in aiding various applications related to medical information, such as patient education, medical education, medical data management, and promoting translational medicine.^6,12^ In relation to stem cell therapy, limited studies have reported on the application of machine learning to process information, mainly to aid scientific or clinical decision-making,^13,14^ such as using gene expression-based machine learning that predicts the genotoxicity of viral vectors for stem cell-based gene therapy.^14^ However, the effectiveness of ChatGPT as an artificial intelligence tool in helping to disseminate information on stem cell therapy has not been studied. To bridge this gap, the present study evaluates for the first time the information obtained by the general public on stem cell therapy through a Google web search compared to ChatGPT.

## Materials and Methods

### Search Strategy

A Google Web Search was conducted on April 18, 2023 using a Google Chrome browser (Version 112.0.5615.138, Beijing, China) that had been clean-installed to minimize the effects of individualized search algorithms. The browser was cleared of all browsing history, cookies, and other site data, as well as cached images, files, and sponsored sites and advertisements. The data retrieval involved searching for the term “stem cell therapy” and extracting the first 20 frequently asked questions (FAQs) along with the source website for each question. The “People also ask” function in Google Web Search was used to identify the questions, and additional questions were obtained by clicking on each question. In the process of selecting the TOP questions, we found that once more than 20 samples were selected, the meanings expressed by the questions in the search were often repeated with those of previous questions. Therefore, in order to ensure the representativeness of the selected samples, only the top 20 were selected.

### Inclusion and Exclusion Criteria

Inclusion criteria for this study involved questions containing the term “stem cell therapy” or “stem cell treatment,” while exclusion criteria were duplicate questions and questions that did not include the defined terms (eg, “What are risks of stem cells?”). The institutional Review Board deemed this study exempt from approval.

### Outcomes

The following statement was input into ChatGPT: “Perform a Google search with the search term ‘stem cell therapy’ and record the 20 most popular questions related to the search term.” The top 20 questions and answers provided as output by ChatGPT were recorded. In addition, the top 10 questions identified by the Google search were input separately into ChatGPT, and the answer for each question was recorded accordingly.

### Comparison Analysis

Classification categories used for comparison analysis are presented in Table 1. The questions were categorized into question topics: Fact, Policy, and Value, as previously reported in the literature using a modification of the Rothwell system.^15^ The questions were also subcategorized into 11 topics relevant to stem cell therapy/treatment: Education, Accessibility, Timeline of Recovery, Restrictions, Technical Details, Cost, Indications/Management, Risks and Complications, Pain, Longevity, and Evaluation of Surgery. Websites were categorized into the following groups in accordance with previous studies^15,16^: commercial, academic, medical practice, single surgeon personal, government, and social media (Table 1). Data categorization was performed by 2 independent reviewers and discrepancies were addressed by consulting a third party.

**Table 1.** Classification rules of questions and websites.

Question topics categorized by Rothwell’s classification	
Fact	Asks whether something is true and to what extent, objective information
Education	Educational, cannot be classified into other subcategories
Accessibility	Where, how can patients get the treatment
Timeline of recovery	Length of time for recovery milestones
Restriction	Restrictions to activity or lifestyle during recovery
Technical details	Details of the treatment procedure
Cost	Cost of treatment and/or rehabilitation
Policy	Asks whether a specific course of action should be taken to solve a problem
Indications/management	Therapy indications and alternatives, postoperative management, timing of therapy
Risks/complication	Risks/complications before, during or after treatment, including rehabilitation period
Value	Asks for evaluation of an idea, object, or event
Pain	Related to the timing, severity, and management of pain
Longevity	Longevity of stem cell therapy
Evaluation of treatment	Successfulness, seriousness, or invasiveness of stem cell therapy
Website categorization	
Commercial	Organizations that provide public health information, including medical device/manufacturing/pharmaceutical companies and news outlets
Academic	Universities, academic medical centers, or academic societies
Medical practice	Local hospitals or medical groups without clear academic affiliation
Government	Websites maintained by a national government
Social media	Blog, internet forms, support groups, and non-medical organizations designed for information and video sharing
Single doctor practice	Personal websites maintained by individual surgeons

## Results

**Figure 1.**
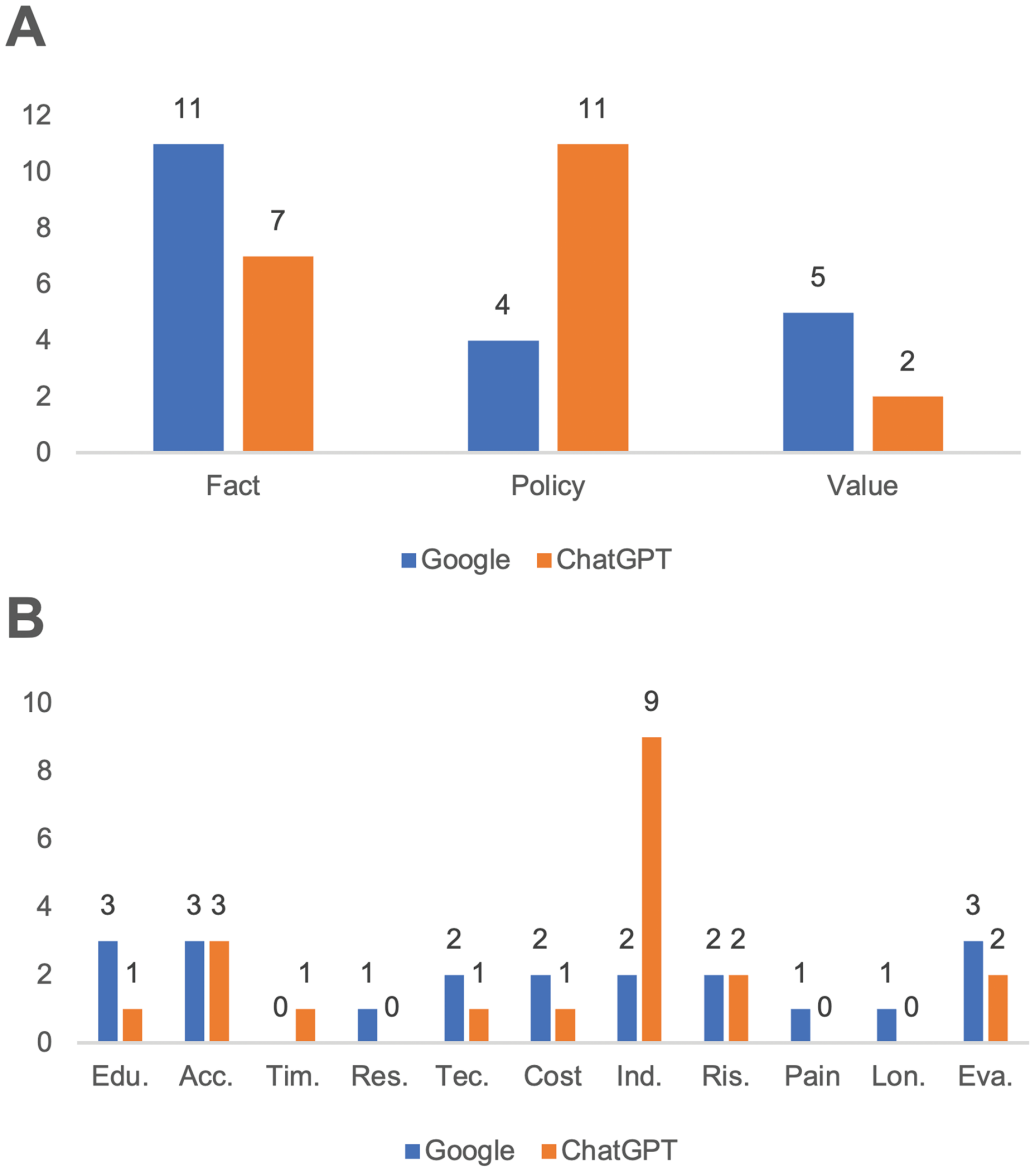
Type distribution of top questions: (**A**) Type distribution of 20 top questions: Google provided 11 questions of the fact class, 4 of the policy class, and 5 of the value class. In contrast, ChatGPT offered 7, 11, and 2 questions for each respective category. (**B**) Subtype distribution of 20 top questions: Google provided 10 question subtypes, while ChatGPT had 9 out of 20 subcategories focused on indication/management. Edu., Acc., Tim., Res., Tec., Ind., Ris., Lon., and Eva. in the chart referred to education, accessibility, timeline of recovery, restrictions, technical details, indications/management, risks/complication, longevity, and evaluation of treatment, respectively.

The top 20 FAQs from Google comprised 11 fact, 4 policy, and 5 value inquiries, while the top 20 questions from ChatGPT comprised 7 fact, 11 policy, and 2 value questions (Table 2, Fig. 1A). The subtypes of the top 20 FAQs from Google were evenly distributed among 10 subcategories, while those from ChatGPT consisted of 8 subcategories, with Indication/Management being the most prevalent subcategory accounting for 9 of the 20 questions (Fig. 1B).

**Table 2.** Top 20 Q&As from Google Web Search.

	Question	Answer
1	What does stem cell therapy do? (fact, education)	Stem cell therapy, also known as regenerative medicine, promotes the repair response of diseased, dysfunctional, or injured tissue using stem cells or their derivatives. It is the next chapter in organ transplantation and uses cells instead of donor organs, which are limited in supply (academic)
2	How successful is stem cell therapy? (value, education)	The success rates of stem cell therapy can vary depending on the type of treatment, the disease or condition being treated, and the stage of the disease. In general, stem cell therapy is considered a safe and effective treatment option for many conditions, and many clinical trials have shown promising results (medical practice)
3	What is the average cost of stem cell therapy? (fact, cost)	The average stem cell therapy cost can range anywhere between $5000 and $50 000. Patients must do their research and ask as many questions, as they can before financially committing to treatment (medical practice)
4	Is stem cell therapy safe? (policy, risks)	Multiple peer-reviewed studies have found mesenchymal stem cell therapy a safe procedure. In addition, over the course of DVC Stem’s IRB-approved clinical study, there have been no reports of any long-term adverse side effects from treatment (medical practice)
5	What are the 4 types of stem cell therapy? (fact, technical details)	ASCs include hematopoietic stem cells (HSCs), skin stem cells (SSCs), neural stem cells (NSCs), and mesenchymal stem cells (MSCs) (government)
6	Is stem cell therapy FDA approved? (fact, accessibility)	Currently, the only stem cell treatments approved by the Food and Drug Administration (FDA) are products that treat certain cancers and disorders of the blood and immune system (government)
7	What are the side effects of stem cell therapy? (policy, risks)	Side effects include: mouth and throat pain, nausea and vomiting, infection, bleeding and transfusions, interstitial pneumonitis and other lung problems, graft-versus-host disease, hepatic veno-occlusive disease (VOD), and graft failure (academic)
8	Is stem cell therapy permanent? (value, longevity)	As the developed cells are injected into the site, they can regenerate new, healthy cells and tissue. These healthy cells may provide you the strength and stability needed to eliminate your discomfort entirely. These results may last a lifetime, depending on your injury, or condition (medical practice)
9	Which country is most advanced in stem cell therapy? (fact, accessibility)	Countries like Japan and Singapore are both seen as leaders in stem cell therapies and, although they might not have the outputs of China, are internationally recognized for the work they continue to do in the field (commercial)
10	What are 3 advantages of stem cell therapy? (value, evaluation)	Studies have discovered that stem cell therapy can help enhance the growth of new healthy skin tissue, enhance collagen production, stimulate hair development after incisions or loss, and help substitute scar tissue with newly developed healthy tissue (medical practice)
11	Is stem cell therapy painful? (value, pain)	Stem cell therapy procedures are not very painful at all. I numb the area to make it as comfortable as possible. And in the event a patient is feeling discomfort, I will add additional numbing medicine to the area to contain it (medical practice)
12	What is the life expectancy of stem cell therapy? (value, evaluation)	Patients who have survived for at least 5 years after hematopoietic cell transplantation without recurrence of the original disease have a high probability of surviving for an additional 15 years, but life expectancy is not fully restored (government)
13	Who pays for stem cell therapy? (fact, cost)	Original Medicare and Medicare Advantage plans both cover certain types of approved stem cell therapy. Medicare parts A and B, also known as original Medicare, provide coverage for approved stem cell treatments and the associated out-of-pocket costs (social media)
14	Where is the best stem cell therapy in the world? (fact, accessibility)	The presentations include: Best Stem Cell Therapy Hospitals in The US Stem Cell Institute, Panama. Enquire Now. Stem Cell Institute is one of the best stem cell hospitals in the world. GIOSTAR. Enquire Now. GIOSTAR is among the leading stem cell therapy providers in the world. Mayo Clinic. Enquire Now. OHSU Knight Cancer Institute (social media)
15	Who is a good candidate for stem cell therapy? (policy, indication)	Patients who have a single joint or cartilage issue that is otherwise in good health may respond well to stem cell therapy, as it works best in healthy people. You can get all the facts about stem cell therapy and have your questions and concerns answered during your consultation (single doctor practice)
16	Why is the FDA against stem cell therapy? (fact, education)	These unproven, unregulated stem cell treatments carry significant risk. The risks range from administration site reactions to dangerous adverse events. For example, injected cells can multiply into inappropriate cell types or even dangerous tumors (government)
17	What not to do after stem cell treatment? (fact, restrictions)	Avoid any forceful rotation or manual manipulation. Remember that good healing during the first 2 months after the procedure will give you the best chance for success. The cells are fragile, and you need to be cautious that you do not overload them or cause too much stress or shearing on them (medical practice)
18	What is an example of stem cell therapy? (fact, education)	The best-defined and most extensively used stem cell treatment is hematopoietic (or blood) stem cell transplantation, for example, bone marrow transplantation, to treat certain blood and immune system disorders or to rebuild the blood system after treatments for some kinds of cancer (academic)
19	What is the most common use of stem cell therapy? (policy, indication)	It is used to treat patients with cancers and disorders that affect the blood and immune system. Stem cell-based therapies for all other conditions are still experimental (academic)
20	Is stem cell therapy a transplant? (fact, technical details)	A bone marrow/stem cell transplant is a medical procedure by which healthy stem cells are transplanted into your bone marrow or your blood. This restores your body’s ability to create the red blood cells, white blood cells, and platelets it needs (academic)

**Figure 2.**
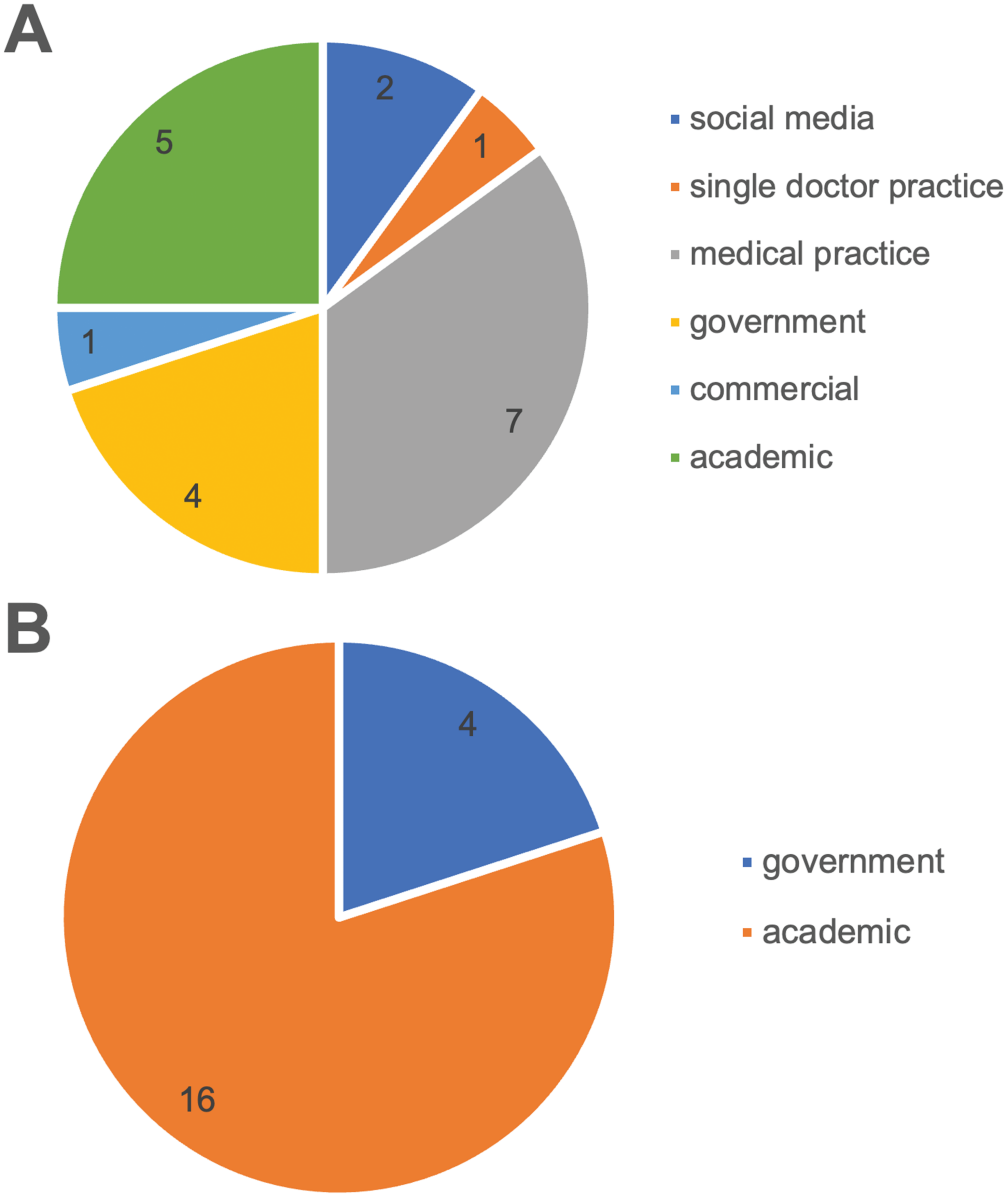
Type distribution of sources of the answers to top 20 questions: (**A**) The answers provided by Google mainly came from medical practice (7/20), academic (5/20), and government (4/20). (**B**) The answers provided by ChatGPT came from academic (16/20) and government (4/20).

Comparing the sources used by Google and ChatGPT to formulate responses to their 20 most popular questions, it was found that Google’s answers were sourced from 5 categories: medical practice, academic, government, social media, and commercial and single doctor practice (Supplementary Table S1). The top 3 sources were medical practice, academic, and government (Fig. 2A). On the other hand, ChatGPT’s answers were primarily derived from academic and government sources, with academic being the dominant source (Fig. 2B).

**Figure 3.**
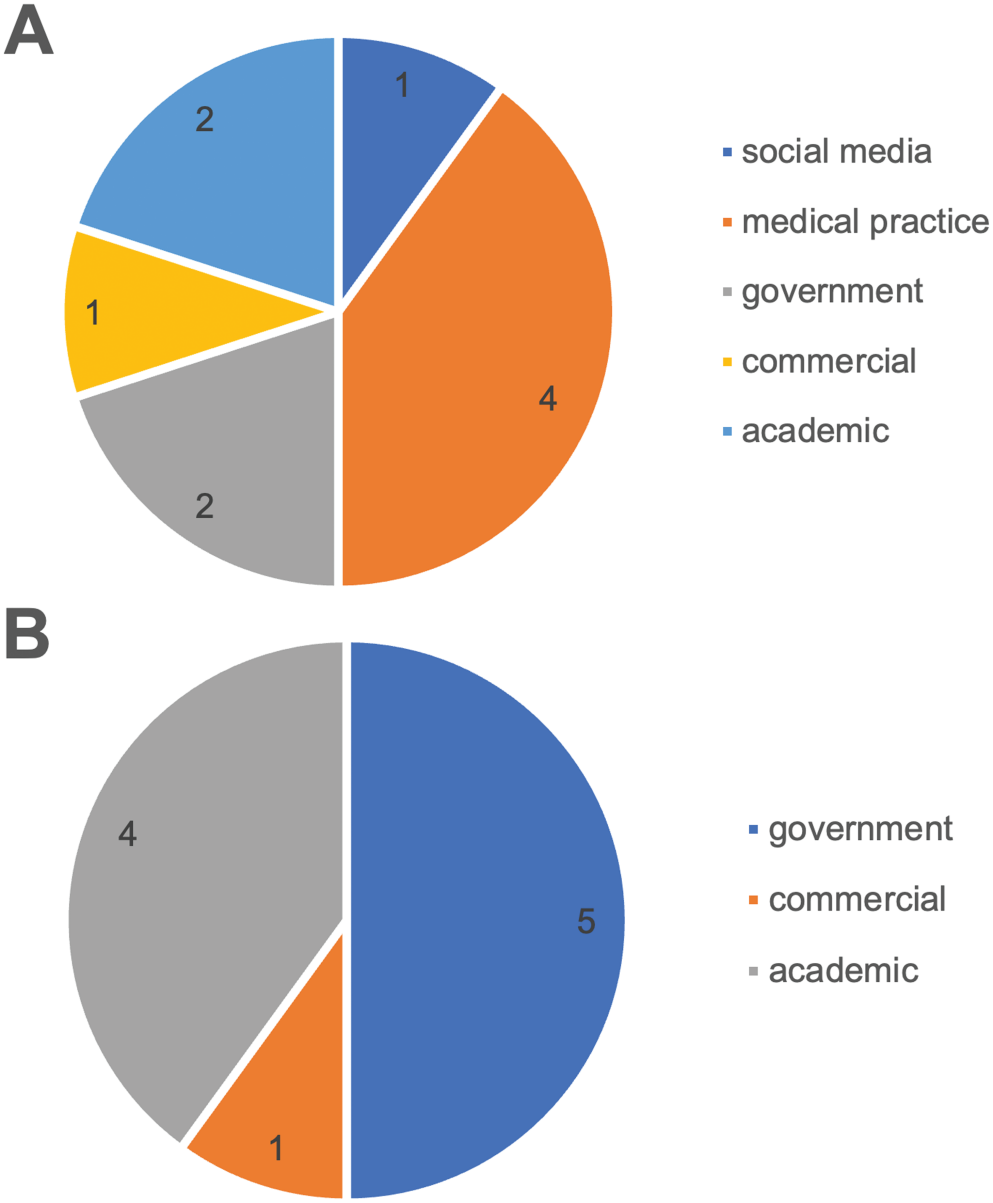
Type distribution of sources of the answers to top 20 questions of Google: (**A**) The answers provided by Google came from medical practice, academic, government, commercial, and social media. (**B**) The answers provided by ChatGPT came from government, academic, and commercial.

Answers by Google and ChatGPT, respectively, to the top 10 questions identified from the Google search were then compared (Supplementary Table S2). It appeared that ChatGPT’s answers were more oriented to government and academic sources, with government accounting for 50% of the answers and academic accounting for 40%. In contrast, Google’s answers came from 5 different sources and did not always have a government or academic focus (Fig. 3A and 3B).

Google Search, a powerful tool for retrieving information, presented fragmented results from various sources (Table 2). In contrast, ChatGPT offered detailed and specific responses that often include medical facts, background information, and referenced data (Supplementary Table S1). This made ChatGPT a valuable resource for in-depth medical inquiries.

## Discussion

It was already reported 10 years ago that more than a third of individuals in the US have turned to the Internet to self-diagnose a health problem, with slightly more than half of those people subsequently discussing their findings with a medical practitioner.^17^ Given a plethora of information now available through the internet on emerging medical treatments such as stem cell therapy, it is more important now than ever that patients can quickly access accurate electronic information on treatment safety and efficacy. Our study suggested that on the topic of stem cell therapy, ChatGPT may have specific advantages in patient education and dissemination of professional information compared to Google. For instance, ChatGPT’s responses to the same questions were mostly drawn from academic and government sources, while Google’s responses were drawn from more variable sources including those that are at higher risk of providing misinformation such as commercial and social media. In addition, Google’s responses may not incorporate information from scientific databases such as PubMed, unlike ChatGPT, which may provide better access to professional answers for nonacademic individuals as well as a better filter for false or misleading information. However, it is important to note that ChatGPT’s answers are still general in nature and at this point in time cannot replace an individual’s critical analysis of academic websites or information sources, particularly for those who are scientifically or medically trained.

While stem cell therapy has been gaining increasing popularity in the last 2 decades, the global market has seen a dramatic increase in commercial entities advertising unproven stem cell interventions. The stem cells market is facing a current dilemma of rapid growth coupled with insufficient regulatory protection and incomplete ethical review.^18^ Some stem cell clinics advertise cell therapy treatments on their website with general statements of efficacy while lacking critical information, such as whether the technology has obtained regulatory approval for clinical application, leaving patients at risk of choosing an unapproved, or even unsafe stem cell product.^19^ To combat this, scientists and clinicians have an important role in advocating for patients and raising public awareness to resist premature and uncontrolled commercialization of stem cell interventions.^20,21^ Our research suggested that ChatGPT might be more effective than Google in identifying and utilizing authoritative sources of information on the applications of stem cell therapy. ChatGPT may, therefore, serve as a valuable tool for patient education on this topic and preventing potential misuse of the treatment. ChatGPT has the potential to enhance decision making in the field of stem cell therapy by integrating relevant information and assisting in making rational decisions. It can provide convenient retrieval of information, making it a valuable tool for patients.

ChatGPT’s communication style, which simulates human language and interaction, has potential to enhance doctor-patient communication.^22^ ChatGPT uses a language simulation model that provides outputs similar to common everyday language, thereby supplying information in a form that may be more acceptable or easily understood by the general public compared to the potentially inconsistent logic or statements frequently present in information found on websites. This may help reduce the likelihood of patients being misled by vague or exaggerated information provided by advertising websites.

Despite its potential advantages, ChatGPT is inadequate for use as a sole source of medically related information or a substitute for academic sources. The current accuracy of ChatGPT’s responses involving such information is questionable due to a large amount of undisclosed training materials. For instance, a study indicated that the accuracy rate of ChatGPT in a medical subspecialty question bank is less than 60%.^23^ Moreover, while the accuracy of information provided by ChatGPT may be improved with increased training, artificial intelligence is unable to offer personalized advice based on individual conditions or local circumstances of the patient’s region of residence.^24^

Other issues worth noting are the ethical and legal concerns raised over the use of ChatGPT. These include its possibility in generating false information, the risk of private information being disclosed,^25^ and variations in its performance across different regions and languages.^26^ The accuracy of information provided by ChatGPT is crucial for clinicians and patients in decision-making and for patients in prediagnosis education, including in the area of stem cell therapy. Our study emphasizes the potential benefits of ChatGPT in providing professional answers to medically related questions on a specific topic area, but it is important to acknowledge the possibility of ChatGPT providing false information depending on the training materials used.

In the field of health communication and medical information dissemination, our methodology plays a vital role in understanding the impact of web-based AI tools on public health decisions and behaviors. By studying the influence of search engine rankings and personalized content recommendations, researchers can explore how these factors shape individuals’ choices regarding health-related information, vaccination decisions, and adherence to medical guidelines. This approach offers valuable insights into the effects of AI-driven information dissemination on public health outcomes. In addition, it would be interesting to compare the results obtained using ChatGPT with those obtained from other search engines or data sources. Our study has some limitations that need to be carefully considered. First, we only considered a limited number of questions, selecting the top 20 FAQs, while a larger number of questions may help improve the accuracy of the results. Second, we attempted to retrieve questions with numerical answers to compare the accuracy between Google and ChatGPT. However, patients are generally more interested in questions focused on treatment efficacy and applicability, and numerical answers were rare among the popular questions. Third, our study only compared the types of questions and the types of answer sources, without quantifying or comparing the accuracy and utility of answers provided by Google and ChatGPT. The second and third limitations meant that a quantitative comparison between Google and ChatGPT could not be performed. Moreover, our study was unable to compare Google Search and ChatGPT in terms of language/region and other factors, indicating the need for further comparative studies involving different regions and language samples. It is important to note that feedback may be influenced by the source language, which can vary across regions, cultures, and communities. Nevertheless, our study has provided interesting findings on the utility of ChatGPT in answering medically related questions and point to the need for more comprehensive, quantitative studies on its applications in medical practice or the dissemination of medical information.

## Conclusion

ChatGPT has potentially stronger capabilities in patient education and providing accurate information on stem cell therapy compared to Google, by predominantly drawing from academic and government sources. However, ChatGPT currently provides generalized responses that lack critical analysis as well as individualized or specialized knowledge. This may improve with continuous training, and a quantitative comparison with web-based search engines such as Google is warranted in future studies. Given the absence of comparative studies on various languages, regions, and cultures in the present study, it is advisable to conduct subsequent comparative studies to explore the distinctions between ChatGPT and Google Search, taking into account relevant factors.

## Supplementary Material

szad074_suppl_Supplementary_Tables

## Data Availability

The data underlying this article will be shared on reasonable request to the corresponding author.
