# Supplementary material for: Using A Google Web Search Analysis to Assess the Utility of ChatGPT in Stem Cell Therapy
Source: Stem Cells Transl Med. 2023 Nov 4;13(1):60–8. doi: 10.1093/stcltm/szad074 (PMC10785216; doi:10.1093/stcltm/szad074)
Supplement: szad074_suppl_Supplementary_Tables [file szad074_suppl_supplementary_tables.docx]

**Table S1** Top 20 Q&As from ChatGPT

|  | Question | Answer |
| --- | --- | --- |
| 1 | What is stem cell therapy?  (fact, education) | Stem cell therapy is a type of regenerative medicine that involves the use of stem cells to repair or replace damaged cells and tissues in the body. Stem cells are undifferentiated cells that have the ability to differentiate into specialized cells and can self-renew to produce more stem cells.  According to the National Institute of Health (NIH), stem cells can be used in a variety of ways for therapeutic purposes, such as to replace or repair damaged tissues, to stimulate the body's own repair mechanisms, or to modulate the immune system. Stem cells can be obtained from various sources, including embryonic stem cells, adult stem cells, and induced pluripotent stem cells.  Stem cell therapy has shown promise in treating a range of conditions, including cancer, autoimmune diseases, neurological disorders, and cardiovascular disease. However, much research is still needed to fully understand the potential of stem cells and how they can be used effectively in clinical practice. (government) |
| 2 | How does stem cell therapy work?  (fact, technical details) | Stem cell therapy works by using stem cells to replace or repair damaged or diseased tissues in the body. Stem cells have the ability to differentiate into specialized cells and can self-renew to produce more stem cells.  According to Mayo Clinic, stem cells can be obtained from various sources, including bone marrow, adipose tissue, and umbilical cord blood. Once obtained, the stem cells can be processed and prepared for transplantation.  The stem cells can then be injected into the affected area or administered intravenously, where they migrate to the damaged tissue and begin to differentiate into the specific cell types needed for repair. Additionally, stem cells can secrete growth factors and other molecules that promote tissue regeneration and modulate the immune system.  Stem cell therapy has shown promise in treating a variety of conditions, including neurological disorders, cardiovascular disease, and autoimmune diseases. However, more research is needed to fully understand the potential benefits and risks of stem cell therapy. (academic) |
| 3 | What conditions can be treated with stem cell therapy?  (policy, indication) | Stem cell therapy has shown promise in treating a wide range of conditions, including:  Blood disorders: Stem cell transplants can be used to treat leukemia, lymphoma, and other blood cancers, as well as certain non-cancerous blood disorders such as sickle cell anemia and thalassemia.  Neurological disorders: Stem cells have been used to treat conditions such as Parkinson's disease, multiple sclerosis, and spinal cord injuries.  Autoimmune diseases: Stem cell therapy has been used to treat autoimmune diseases such as lupus, rheumatoid arthritis, and Crohn's disease.  Cardiovascular disease: Stem cells have been used to treat heart attack, heart failure, and peripheral artery disease.  Orthopedic injuries: Stem cells have been used to treat injuries such as torn tendons, ligaments, and cartilage. (academic) |
| 4 | Is stem cell therapy safe?  (policy, risks) | The safety of stem cell therapy depends on a number of factors, including the source of the stem cells, the method of delivery, and the condition being treated. While stem cell therapy has shown promise in treating a variety of conditions, there are also potential risks and complications.  According to the International Society for Stem Cell Research (ISSCR), the use of untested or unproven stem cell treatments can be risky and may result in serious harm to patients. The ISSCR recommends that patients considering stem cell therapy should seek out qualified medical professionals who are using approved stem cell therapies that have been tested in clinical trials.  Additionally, the U.S. Food and Drug Administration (FDA) has issued warnings about the potential risks of stem cell therapies that have not been approved by the FDA. These unapproved therapies may be marketed as "stem cell clinics" and may use stem cells obtained from untested sources, such as fat or amniotic fluid.  While the use of stem cells in approved clinical trials is generally considered safe, there is still much research needed to fully understand the long-term safety and efficacy of stem cell therapy. (academic) |
| 5 | What are the benefits of stem cell therapy?  (value, evaluation) | Stem cell therapy has shown promise in treating a variety of conditions and has several potential benefits, including:  Tissue repair and regeneration: Stem cells can differentiate into specific cell types and promote tissue repair and regeneration.  Reduced inflammation: Stem cells can secrete molecules that modulate the immune system and reduce inflammation. Minimal invasiveness: Many stem cell therapies are minimally invasive and do not require surgery.  Fewer complications: Compared to traditional treatments, stem cell therapy may result in fewer complications and side effects.  Personalized treatment: Stem cell therapy can be customized to the individual patient, taking into account their unique medical history and condition. (government) |
| 6 | What are the risks of stem cell therapy?  (policy, risks) | While stem cell therapy has shown promise in treating a variety of conditions, there are also potential risks and complications. Some of the risks associated with stem cell therapy include:  Tumor formation: There is a risk that stem cells may form tumors or cause existing tumors to grow.  Immune rejection: Stem cells may be recognized as foreign by the immune system and rejected, leading to graft versus host disease.  Infection: There is a risk of infection from stem cell transplantation, particularly if the stem cells are obtained from a donor.  Bleeding and other complications: Stem cell transplantation can lead to bleeding and other complications, particularly if it involves high-dose chemotherapy.  Ethical concerns: There are also ethical concerns regarding the use of embryonic stem cells, which involves the destruction of human embryos. (academic) |
| 7 | How much does stem cell therapy cost?  (fact, cost) | The cost of stem cell therapy can vary widely depending on the type of therapy, the condition being treated, the location of the clinic, and other factors. Stem cell therapy can range from a few thousand dollars to tens of thousands of dollars per treatment.  According to the International Society for Stem Cell Research (ISSCR), the cost of stem cell therapy is often not covered by insurance because it is considered an experimental treatment. Patients considering stem cell therapy should be prepared to pay for the treatment out of pocket and should be aware of the potential risks and uncertainties associated with the treatment.  It's important to note that some clinics may offer unproven stem cell treatments that are not backed by scientific evidence. These treatments can be expensive and may not provide any benefit to the patient. (academic) |
| 8 | How long does it take to see results from stem cell therapy?  (fact, timeline of recovery) | The time it takes to see results from stem cell therapy can vary depending on the type of therapy and the condition being treated. Some patients may see improvement in their symptoms within a few weeks, while others may take several months to see a noticeable difference.  According to the National Institutes of Health (NIH), the time it takes to see results from stem cell therapy can depend on several factors, including the type of cells used, the method of delivery, and the patient's overall health and medical history.  It's also important to note that not all patients will respond to stem cell therapy, and some may not see any improvement in their symptoms. (government) |
| 9 | Where can I get stem cell therapy?  (fact, accessibility) | Stem cell therapy is still an experimental treatment and is not widely available. However, there are a growing number of clinics and medical centers that offer stem cell therapy for certain conditions.  If you are interested in pursuing stem cell therapy, it's important to do your research and choose a reputable clinic or medical center that has experience with stem cell therapy and follows ethical and safety guidelines.  You may also want to consult with your primary care physician or specialist to discuss whether stem cell therapy is a viable treatment option for your condition and to obtain a referral to a qualified provider. (academic) |
| 10 | Is stem cell therapy FDA approved?  (fact, accessibility) | There are currently only a few stem cell therapies that have been approved by the United States Food and Drug Administration (FDA). These include treatments for certain blood and immune disorders, such as leukemia and graft-versus-host disease.  However, many stem cell therapies are still considered experimental and have not been approved by the FDA. It's important to be cautious of clinics or providers that offer unapproved stem cell treatments or make exaggerated claims about the effectiveness of the treatment.  The FDA is working to establish clear guidelines for the development and use of stem cell therapies, and is taking action against clinics and providers that offer unproven or unsafe treatments. (government) |
| 11 | What types of stem cell therapy are available?  (fact, accessibility) | There are several types of stem cell therapy available, each with its own unique advantages and disadvantages. Some of the most common types of stem cell therapy include:  Autologous stem cell therapy: This type of therapy involves using a patient's own stem cells, which are harvested and then re-infused back into the patient's body.  Allogeneic stem cell therapy: This type of therapy involves using stem cells from a donor, which are matched to the patient's tissue type to minimize the risk of rejection.  Umbilical cord blood stem cell therapy: This type of therapy involves using stem cells from the blood of a newborn's umbilical cord, which are collected and stored for future use.  Embryonic stem cell therapy: This type of therapy involves using stem cells derived from embryos, which can differentiate into almost any cell type in the body. I  nduced pluripotent stem cell therapy: This type of therapy involves using reprogrammed adult cells, which are induced to differentiate into specific cell types. (academic) |
| 12 | What is the success rate of stem cell therapy?  (value, evaluation) | The success rate of stem cell therapy can vary depending on the type of therapy and the condition being treated. Some studies have reported positive outcomes, while others have found little to no benefit from stem cell therapy.  It's also important to note that stem cell therapy is still considered an experimental treatment, and more research is needed to fully understand its safety and effectiveness.  According to the International Society for Stem Cell Research, the success rate of stem cell therapy can depend on several factors, including the type of cells used, the method of delivery, the timing and frequency of treatment, and the patient's overall health and medical history. (academic) |
| 13 | Can stem cell therapy help with arthritis?  (policy, academic) | Stem cell therapy has shown potential in the treatment of arthritis, particularly osteoarthritis, which is a degenerative joint disease that affects millions of people worldwide.  According to a review article published in the journal Stem Cell Research & Therapy, clinical studies have shown that mesenchymal stem cell therapy can improve symptoms and function in patients with osteoarthritis. These studies have also shown that the therapy is safe and well-tolerated.  However, more research is needed to fully understand the potential benefits of stem cell therapy for arthritis, as well as to optimize the delivery method and dosage of the therapy. (academic) |
| 14 | Can stem cell therapy help with back pain?  (policy, academic) | Stem cell therapy has shown potential in the treatment of back pain, particularly chronic low back pain caused by degenerative disc disease.  According to a review article published in the journal Stem Cells Translational Medicine, preclinical and clinical studies have shown that mesenchymal stem cell therapy can promote tissue regeneration and reduce inflammation in degenerative discs, leading to improvements in pain and function.  However, more research is needed to fully understand the potential benefits of stem cell therapy for back pain, as well as to optimize the delivery method and dosage of the therapy. (academic) |
| 15 | Can stem cell therapy help with knee pain?  (policy, academic) | Stem cell therapy has shown potential in the treatment of knee pain, particularly osteoarthritis of the knee.  According to a review article published in the journal Stem Cells Translational Medicine, clinical studies have shown that mesenchymal stem cell therapy can improve pain and function in patients with knee osteoarthritis. These studies have also shown that the therapy is safe and well-tolerated.  However, more research is needed to fully understand the potential benefits of stem cell therapy for knee pain, as well as to optimize the delivery method and dosage of the therapy. (academic) |
| 16 | Can stem cell therapy help with hair loss?  (policy, academic) | Stem cell therapy has shown promising results in treating hair loss. Stem cells have the potential to differentiate into various cell types, including hair follicle cells, which can promote hair growth. Studies have shown that stem cell therapy can increase hair density and thickness in patients with androgenetic alopecia, a common type of hair loss.  One study published in the Journal of Stem Cells and Regenerative Medicine found that injecting adipose-derived stem cells into the scalps of patients with androgenetic alopecia led to an increase in hair density and thickness after six months. Another study published in the Journal of Cutaneous and Aesthetic Surgery reported similar results with the use of platelet-rich plasma, a substance rich in growth factors that can stimulate hair growth. (academic) |
| 17 | Can stem cell therapy help with multiple sclerosis?  (policy, academic) | There is some evidence to suggest that stem cell therapy may be a promising treatment option for multiple sclerosis (MS). According to a review published in the Journal of Neurology, Neurosurgery & Psychiatry in 2020, hematopoietic stem cell transplantation (HSCT) has been shown to induce remission in patients with relapsing-remitting MS (RRMS) and may be a potential cure for the disease.  However, it's important to note that HSCT is a high-risk procedure and is typically reserved for patients with aggressive forms of MS who have not responded to other treatments. Additionally, more research is needed to fully understand the safety and efficacy of stem cell therapy for MS. (academic) |
| 18 | Can stem cell therapy help with diabetes?  (policy, academic) | Stem cell therapy is a promising treatment option for diabetes. Stem cells have the potential to differentiate into insulin-producing cells, which could help restore normal insulin production in individuals with diabetes. Research in animals and humans has shown that stem cell therapy can improve blood sugar control and reduce the need for insulin injections.  One example of such research is a study published in the journal Stem Cell Research & Therapy in 2018, which found that patients with type 2 diabetes who received stem cell therapy showed significant improvements in their blood sugar levels and insulin resistance compared to those who did not receive the therapy. (academic) |
| 19 | Can stem cell therapy help with heart disease?  (policy, academic) | Stem cell therapy is a promising treatment option for heart disease. Stem cells have the potential to differentiate into heart muscle cells, which could help repair damaged heart tissue and improve heart function. Research in animals and humans has shown that stem cell therapy can improve heart function and reduce the risk of death and hospitalization for heart failure.  One example of such research is a study published in the New England Journal of Medicine in 2017, which found that patients with heart failure who received stem cell therapy had a significant improvement in their left ventricular ejection fraction (a measure of heart function) compared to those who received a placebo. (academic) |
| 20 | Can stem cell therapy cure cancer?  (policy, academic) | Stem cell therapy is not a cure for cancer. In fact, stem cells have been found to play a role in the development and progression of some types of cancer, including leukemia and some solid tumors. While stem cell transplantation can be used as a treatment for some types of cancer, such as leukemia, it is not considered a cure.  One example of such research is a study published in the journal Stem Cells Translational Medicine in 2019, which found that stem cells can promote the growth of certain types of breast cancer cells and increase their resistance to chemotherapy. (academic) |

**Table S2** Answers to top 10 questions of Google Web Search from ChatGPT

|  | Question | Answer |
| --- | --- | --- |
| 1 | What does stem cell therapy do? | Stem cell therapy is a type of medical treatment that uses stem cells to repair, replace or regenerate damaged or diseased tissues or organs in the body. Stem cells have the ability to develop into many different types of cells in the body and can also renew themselves through cell division. Stem cell therapy has shown promise in the treatment of a wide range of medical conditions, including neurological disorders, cardiovascular diseases, autoimmune diseases, blood disorders, orthopedic injuries, and eye diseases. (government) |
| 2 | How successful is stem cell therapy? | Stem cell therapy has shown promise in treating various medical conditions, including some types of cancer, autoimmune diseases, and tissue injuries. However, the success of stem cell therapy depends on several factors, including the type of stem cells used, the method of administration, and the patient's specific condition.  According to the National Institute of Health (NIH), some stem cell therapies have shown significant clinical benefits in treating certain diseases, while others are still in the early stages of research and require more investigation. For example, bone marrow transplants using hematopoietic stem cells have been successful in treating certain types of blood cancers, such as leukemia and lymphoma. However, the use of stem cells for treating other conditions, such as Parkinson's disease and spinal cord injuries, is still experimental and requires further clinical trials.  Overall, while stem cell therapy has shown promise in treating certain medical conditions, its success depends on the specific context of each application, and further research is needed to determine its full potential. (government) |
| 3 | What is the average cost of stem cell therapy? | The cost of stem cell therapy varies widely depending on the type of treatment, the location of the clinic, and the individual patient's needs. In general, stem cell therapy can cost anywhere from a few thousand dollars to tens of thousands of dollars.  According to a 2020 report by BioInformant, a market research firm specializing in stem cell industry analysis, the average cost of stem cell therapy in the United States is approximately $10,000 to $30,000 per treatment, with some treatments costing as much as $50,000 or more.  It's important to note that many stem cell therapies are still experimental and may not be covered by health insurance. Patients should consult with their healthcare providers and insurance companies to determine whether stem cell therapy is a covered treatment option and what costs may be involved. (commercial) |
| 4 | Is stem cell therapy safe? | Stem cell therapy can be generally considered safe, but as with any medical treatment, there are potential risks and side effects. The safety of stem cell therapy depends on several factors, including the type of stem cells used, the method of administration, and the patient's specific condition.  According to the International Society for Stem Cell Research (ISSCR), the risks of stem cell therapy include the potential for the cells to form tumors or unwanted tissue, the risk of infection from the procedure, and the possibility of the cells not working as intended. The ISSCR also notes that some stem cell clinics may make misleading claims or offer treatments that have not been proven effective or safe.  It's essential to undergo stem cell therapy at a reputable clinic and under the guidance of a licensed healthcare professional. Patients should also be aware that the regulatory landscape for stem cell therapy is complex and may vary by country or region. (academic) |
| 5 | What are the 4 types of stem cell therapy? | There are four main types of stem cell therapy, each with its own unique characteristics and potential applications:  1.Autologous stem cell therapy: This involves using stem cells derived from the patient's own body, such as bone marrow or adipose tissue. These cells are typically collected, processed, and then injected back into the patient to help repair or regenerate damaged tissue.  2.Allogeneic stem cell therapy: This involves using stem cells derived from a donor, which may be a family member or an unrelated individual. The donor cells are typically screened to ensure compatibility and then infused into the patient's body to help treat certain medical conditions.  3.Xenogeneic stem cell therapy: This involves using stem cells derived from a different species, such as pigs or cows. While this approach is still experimental, it has the potential to offer a new source of stem cells for medical treatments.  4.Induced pluripotent stem cell therapy: This involves reprogramming adult cells to become pluripotent stem cells, which have the ability to differentiate into different cell types. These cells can then be used for research or potentially for therapeutic purposes. (government) |
| 6 | Is stem cell therapy FDA approved? | Some stem cell therapies have received approval from the U.S. Food and Drug Administration (FDA) for specific medical conditions, but many stem cell therapies are still considered experimental and are not yet approved for widespread use.  The FDA regulates stem cell therapies based on their intended use, the type of cells used, and how the cells are manipulated or processed. The agency has approved several stem cell therapies for specific medical conditions, including bone marrow transplants for certain blood disorders and tissue-engineered products for skin and bone regeneration.  However, the FDA has issued warning letters and taken enforcement action against clinics and companies that market unapproved stem cell products or make false or misleading claims about their safety or efficacy. Patients should be cautious about seeking out stem cell therapies that have not been FDA-approved or that are marketed as a cure-all for a range of medical conditions. (government) |
| 7 | What are the side effects of stem cell therapy? | Stem cell therapy can have potential side effects, although the specific risks and complications depend on the type of treatment and the individual patient's health status. Some of the potential side effects of stem cell therapy may include:  Tumor formation: There is a risk that stem cells can develop into unwanted tissue, including tumors.  Infection: Patients may be at risk of developing an infection at the site of stem cell administration or from the stem cells themselves.  Immune system reactions: In some cases, the body's immune system may recognize transplanted stem cells as foreign and attack them.  Pain and swelling: Some patients may experience pain, swelling, or inflammation at the site of stem cell administration.  Graft-versus-host disease (GVHD): This is a potential complication of allogeneic stem cell transplants, where the donor's immune cells attack the recipient's body.  It's important to note that these side effects are rare, and most patients do not experience any significant complications from stem cell therapy. Patients should speak with their healthcare provider about the potential risks and benefits of stem cell therapy for their specific condition. (academic) |
| 8 | Is stem cell therapy permanent? | The permanence of stem cell therapy depends on the specific treatment and the individual patient's response. In some cases, stem cell therapy can provide long-lasting benefits, while in other cases, the effects may be temporary.  For example, in some cases of bone marrow transplant, the transplanted stem cells can provide a lifelong supply of healthy blood cells. In other cases, such as stem cell treatments for joint pain or certain types of injuries, the effects may be temporary, and the patient may require additional treatments over time to maintain the benefits.  It's also important to note that stem cell therapy is still an evolving field, and researchers are working to develop new and more effective treatments that could provide more lasting benefits. (academic) |
| 9 | Which country is most advanced in stem cell therapy? | There are several countries that are at the forefront of stem cell research and therapy, with significant investments in research and development. Some of the countries that are considered leaders in stem cell therapy include:  United States: The U.S. has a robust stem cell research community, with many leading academic institutions and companies working on developing new stem cell therapies.  Japan: Japan has invested heavily in stem cell research and has established a regulatory framework that allows for the clinical use of induced pluripotent stem cells.  South Korea: South Korea has made significant advances in stem cell research and has a strong track record in producing high-quality research in this field.  China: China has invested heavily in stem cell research and has a large and growing stem cell industry. (academic) |
| 10 | What are three advantages of stem cell therapy? | Stem cell therapy has several potential advantages over traditional medical treatments, including:  Regeneration of damaged tissue: Stem cells have the ability to differentiate into different types of cells and can regenerate damaged or diseased tissue, potentially providing a long-lasting cure.  Reduced risk of rejection: Stem cells can be obtained from the patient's own body (autologous transplantation) or from a closely matched donor (allogeneic transplantation), reducing the risk of rejection by the immune system.  Reduced need for surgery: Some types of stem cell therapy can be administered through injection or infusion, reducing the need for invasive surgery. (government) |
